# Alcohol-Induced Histone Acetylation Reveals a Gene Network Involved in Alcohol Tolerance

**DOI:** 10.1371/journal.pgen.1003986

**Published:** 2013-12-12

**Authors:** Alfredo Ghezzi, Harish R. Krishnan, Linda Lew, Francisco J. Prado, Darryl S. Ong, Nigel S. Atkinson

**Affiliations:** Section of Neurobiology and Waggoner Center for Alcohol and Addiction Research, The University of Texas at Austin, Austin, Texas, United States of America; Rutgers University, United States of America

## Abstract

Sustained or repeated exposure to sedating drugs, such as alcohol, triggers homeostatic adaptations in the brain that lead to the development of drug tolerance and dependence. These adaptations involve long-term changes in the transcription of drug-responsive genes as well as an epigenetic restructuring of chromosomal regions that is thought to signal and maintain the altered transcriptional state. Alcohol-induced epigenetic changes have been shown to be important in the long-term adaptation that leads to alcohol tolerance and dependence endophenotypes. A major constraint impeding progress is that alcohol produces a surfeit of changes in gene expression, most of which may not make any meaningful contribution to the ethanol response under study. Here we used a novel genomic epigenetic approach to find genes relevant for functional alcohol tolerance by exploiting the commonalities of two chemically distinct alcohols. In *Drosophila melanogaster*, ethanol and benzyl alcohol induce mutual cross-tolerance, indicating that they share a common mechanism for producing tolerance. We surveyed the genome-wide changes in histone acetylation that occur in response to these drugs. Each drug induces modifications in a large number of genes. The genes that respond similarly to either treatment, however, represent a subgroup enriched for genes important for the common tolerance response. Genes were functionally tested for behavioral tolerance to the sedative effects of ethanol and benzyl alcohol using mutant and inducible RNAi stocks. We identified a network of genes that are essential for the development of tolerance to sedation by alcohol.

## Author Summary

Alcoholism is a complex condition of compulsive alcohol use that results in devastating physical and social consequences. The development of this affliction is believed to arise in part by homeostatic adaptations in the brain that lead to the development of alcohol tolerance and dependence. These adaptations are strongly influenced by a great number of genetic and environmental conditions. Identifying the relevant factors that define alcohol tolerance and dependence has been a major objective of neurobiology research for many decades. Here we use a novel genomic approach that exploits the analysis of epigenetic modifications and the power of Drosophila genetics to identify a network of genes with a potential role in the neuroadaptations that lead to alcohol tolerance. Gene-expression profiling and subsequent gene ontology analysis revealed that the group of genes identified here is highly enriched with genes involved in the activity-dependent modulation of synaptic transmission. Because of the strong conservation of regulatory gene networks between Drosophila and mammals, we believe that the network identified here will serve as a powerful guide for the identification of the regulatory events that lead to human alcohol tolerance.

## Introduction

Drug tolerance and dependence are two key components in the development of drug addiction. These drug responses are believed to arise from common homeostatic adaptations in the brain that oppose the effects of the drug [1], [2]. Tolerance in particular is a reduction in drug sensitivity in response to prior drug exposure. While this adaptation ameliorates the effects of intoxication, it often outlives the intoxicated state to produce symptoms of withdrawal. Not only are these symptoms indicative of physiological dependence but also both tolerance and withdrawal appear to act in a feed-forward kindling-like manner to deepen the addicted state [3]. Therefore, understanding the mechanisms that underlie tolerance to alcohol is of central importance for understanding alcoholism.

Modulation of gene expression has emerged as an important mechanism in the development of brain adaptations that produce drug-induced changes in behavior [4]. In particular, epigenetic histone modifications have become central to our understanding of drug abuse. They serve as a molecular memory of previous drug experiences that leads to altered responsivity during future drug exposures. Drug-induced changes in histone acetylation, for example, have been shown to be a major component in the long-term adaptation that leads to tolerance to alcohols in both Drosophila and mammals [5], [6]. Therefore, a genomic survey of histone acetylation may be instrumental in identifying genes whose coordinate regulation mediates drug-induced adaptations.

While high-throughput expression studies have proven successful for the discovery of differences in gene expression that define cell types, the same methods have been less successful in the identification of genes that underlie drug-induced changes in behavior [7]. We believe that the major constraint impeding progress is that genes important for a specific drug response are obscured by the overwhelming abundance of changes in gene expression observed in response to drug exposure. Most of these changes may not produce any meaningful contribution to the behavior under study. This limitation has led the field to focus largely on meta-analysis of genomic data, but even extensive meta-analysis can result in an unwieldy number of gene candidates [8].

To circumvent this problem, we used a novel genomics-based epigenetic approach to specifically identify genes that underlie functional tolerance to alcohol sedation in Drosophila. This approach is based on the observation that some chemically distinct alcohols produce mutual cross-tolerance in a mechanistically related manner. We reasoned that the genes that show related patterns of histone acetylation in response to both drugs are likely to be involved in producing the common behavioral response, while genes that are unimportant for the shared behavior are unlikely to display similar histone acetylation profiles in response to these chemically distinct drugs.

In Drosophila, tolerance to ethanol and to the solvent anesthetic benzyl alcohol has been shown to develop after a single exposure to the either drug [9]–[11]. Furthermore, both drugs were shown to induce cross-tolerance to each other, indicating that they share a common mechanism for the development of tolerance [12]. Using genetic analysis, we have previously demonstrated that the mechanism of tolerance to these drugs involves a drug-induced upregulation of the BK-type Ca^2+^-activated K^+^ channel encoded by the *slo* gene. We showed that in the nervous system, the Drosophila *slo* gene responds to solvent intoxication with a programmed transcriptional response whose progression is mediated by a dynamic increase in histone H4 acetylation [5], [13]. In turn, increased *slo* gene expression acts as a neural excitant that, upon subsequent exposure, counters the sedating effect of the drug to produce tolerance. However, after drug clearance, the persistent increase in channel activity reduces the animal's seizure threshold, giving rise to a withdrawal phenotype [14], [15].

In order to identify new genes that are co-regulated with *slo* and, like *slo*, participate in the neuroadaptations behind the development of drug tolerance, we conducted genome-wide surveys of histone acetylation changes produced in response to either ethanol or benzyl alcohol. A subset of the genes with similar responses to both drugs was evaluated by mutant analysis to functionally test their role in producing alcohol tolerance. Here we report the identification of a highly correlated network of genes with direct roles in the modulation of neural activity that are essential for the development of tolerance to sedation by alcohol.

## Results

### Alcohol-induced changes in histone acetylation

**Figure 1:**
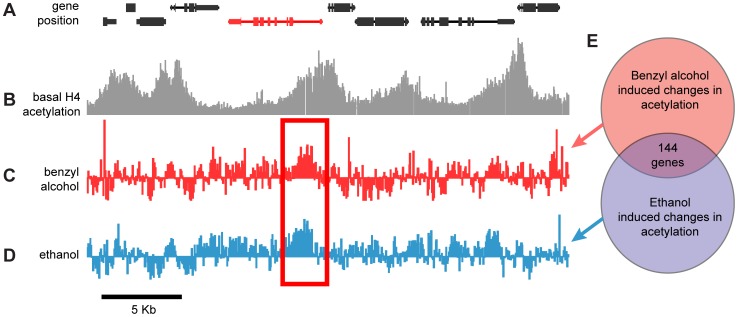
Genome-wide, drug-induced changes in H4 acetylation. **A**) Annotated gene map of a 30 kb representative region of the Drosophila chromosome 3R. The coding region of depicted genes is shown as connected boxes. Genes in the top row are transcribed from the plus (+) strand (left to right); genes in the bottom row are transcribed from the minus (−) strand (right to left). **B**) Histone acetylation profile of chromatin isolated from wild-type fly heads. Plot shows histone H4 acetylation levels of untreated control flies across the same chromosomal region displayed in (**A**). Bars represent the normalized IP/input ratios of fluorescently labeled chromatin signals detected by a single DNA tilling array. **C–D**) Difference plot showing the changes in histone H4 acetylation between control flies and benzyl alcohol-treated flies (**C**) or control flies and ethanol-treated flies (**D**) across the same chromosomal region displayed in (**A**). Bars are log2 values of the normalized IP-treated/IP-control ratios of fluorescently labeled chromatin signals detected by a single DNA tilling array. The red rectangle highlights a representative example of a statistically significant drug-induced acetylation spike shared by both drug treatments. The depicted red gene identifies the closest gene loci associated with the drug-induced acetylation spike. **E**) Diagrammatic representation of the overlap between the cohorts of genes with significant changes in acetylation induced by benzyl alcohol, ethanol or both.

To measure histone acetylation across the fly genome, we performed genomic surveys of histone H4 acetylation (H4Ac) using the chromatin-immunoprecipitation assay (ChIP–chip). Anti-H4Ac immunoprecipitated chromatin and the corresponding “input” chromatin were hybridized to NimbleGen two-color Drosophila DNA tiling arrays. A representative snapshot of the acetylation profile of a 30 Kb region of chromosome 3R (3R:1,406,00..1,436,000) obtained from an untreated control sample is shown in Figure 1. Genes in this region are shown in figure 1A while the respective acetylation profile across the same region is shown in figure 1B. Peaks demarcate highly acetylated regions. In most cases, acetylation peaks overlapped the transcriptional start site of annotated genes, sometimes covering the entire coding region of genes. The histone H4 acetylation “landscape” closely resembled the histone H4 acetylation patterns reported by the modENCODE project, even though we used adult heads instead of whole flies. The veracity of the ChIP–chip data was further confirmed by real-time PCR for 10 unique loci across the fly genome from three independent control chromatin samples (Supporting Figure S1). We chose to survey histone acetylation as a way to monitor gene activation rather than directly measuring changes in mRNA abundance because we wanted to have a strong focus of transcriptional regulation. Changes in mRNA abundance are often produced by the specific regulation of message stability. However, changes in histone acetylation are the direct products of transcription co-factors such as histone acetyl-transferases (HATs) or histone deacetylases (HDACs) that often associate with transcription factors to initiate or prevent transcription. Hence histone acetylation more directly reflects transcription activation state.

To specifically identify drug-induced changes in H4Ac, we hybridized anti-H4Ac immunoprecipitated chromatin from the heads of drug-treated and mock-treated flies in a single two-color Drosophila DNA tiling array. With this approach, changes in the magnitude of H4 acetylation between the control and drug-treated animals generate *difference peaks*. Figures 1C and 1D show the difference peaks generated by benzyl alcohol or ethanol, respectively, for the same region of chromosome 3R. Statistically significant peaks in the difference plots, from two biological replicates, were identified by statistical comparison to a randomized sample using a FDR cutoff <0.05 (Supporting Figure S2). Genes associated with each peak were subsequently identified by proximity after mapping the peaks to the annotated Drosophila genome. While each drug produced significant changes in over 1500 gene loci, only a subset of 144 genes (∼10%) were found in common between the two drugs (Figure 1E). We hypothesized that this intersection will be highly enriched for genes important for functional tolerance, a shared response to both drugs. To reduce the complexity of the analysis, only genes that increase acetylation were examined in this study. A complete list of these genes, including full gene ontology information, is displayed in the accompanying supporting material (Supporting Datasets S1 and S2).

### Gene-expression correlation analysis and gene clustering

An attractive hypothesis is that the genes identified here are co-regulated in an activity-dependent manner and are involved in common processes in the cell. One way to determine similarities between groups of genes is to perform gene annotation clustering analysis, which is based on molecular function or biological process. However, this analysis does not take into account correlated transcriptional activity between the genes. To overcome this limitation, we performed a gene-expression profile analysis to enhance the gene ontology analysis.

**Figure 2:**
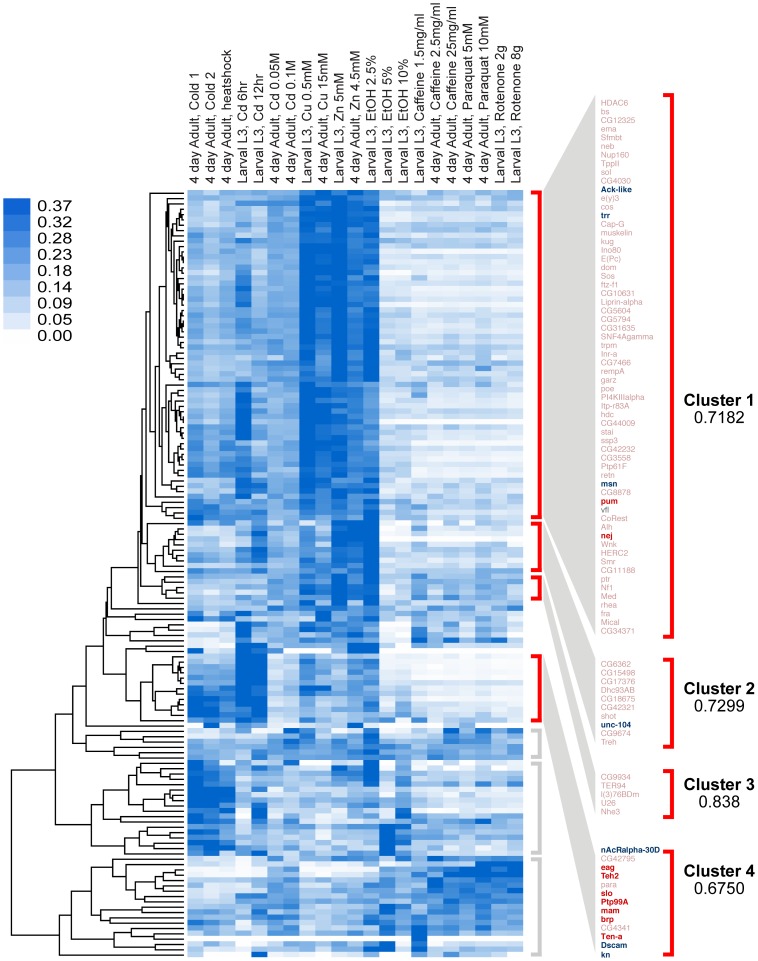
Clustering analysis by gene-expression patterns of genes identified in this study. Genes were clustered by Pearson correlation analysis of their mRNA expression patterns induced by 21 different environmental conditions. Shades of blue in heat map denote gene-expression levels for each condition, normalized for each gene using the sum of squares of all conditions (white is lowest, dark blue highest). After clustering, genes segregate into seven distinct clusters, four of which are highly correlated (r>0.65). These clusters are denoted by red brackets. Clusters with low or no correlation (r<0.3) are denoted by gray brackets. In this study, eighteen genes (16 of which fall within the highly correlated clusters, marked in bold) were tested for their role in behavioral alcohol tolerance. Of these, ten (marked in red) significantly reduced tolerance to alcohol, while eight (only six shown) had no effect (marked in blue).

To identify co-regulated groups within the 144 candidate genes, we first organized them into groups with similar patterns of expression. Gene-expression profiles produced by exposing *Drosophila melanogaster* to various chemicals or subjecting them to temperature shock were obtained from the “Transcriptional Profiling of Compound-based treatments of *D. melanogaster* using Illumina poly(A)+ RNA-Seq” data set (collected by the Brenton Graveley laboratory at the University of Connecticut Health Center to the modENCODE Drosophila Transcriptome project [16]). This data set contains gene-expression profiles from different fly populations treated with four different temperature-shock protocols, eight different heavy-metal diets, exposures to three different ethanol concentrations, three different caffeine-treatment protocols, and four different treatments with oxidative stress agents. Gene-expression profiles of the 144 candidates were subjected to unsupervised hierarchical clustering based on Pearson correlation coefficients [17]. We found that the genes segregated into seven distinct clusters. However, only four of these clusters were highly correlated (r>0.65). Figure 2 shows the 144 genes after gene-expression clustering and the members of each highly correlated gene cluster. The similar response to a variety of unrelated treatments suggests that these genes are co-regulated.

Because the genes in each cluster share highly correlated gene-expression patterns, it is expected that genes within each cluster also share common functional roles. To determine if the genes in each Gene-Expression Cluster have related function, we gathered gene ontology information for each gene. We performed gene annotation clustering within the gene groups shown in Figure 2 using the Database for Annotation, Visualization and Integrated Discovery (DAVID) tool [18]. A high percentage of genes within each Gene-Expression Cluster have common molecular functions and/or participate in a common biological process. The top terms in each DAVID cluster (Fisher Exact/EASE Score p-value<0.05) are listed in Table 1. We found Gene-Expression Cluster #1 to be highly enriched for genes associated with transcription regulation, chromatin regulation, and small regulatory proteins (phosphorylation and GTPase activity) amongst others. Gene-Expression Cluster #2 is enriched in adenyl nucleotide binding and microtubule cytoskeleton genes. Gene-Expression Cluster #4 is highly enriched for genes associated with ion channel activity and synaptic membrane proteins. Gene-Expression Cluster #3 did not display significant enrichment for any gene ontology category. Full gene ontology annotation information for all clusters can be found in Supporting Dataset S2.

**Table 1:** Gene ontology annotations for highly correlated gene clusters.

Expression Cluster	Gene Ontology Term	Count (%)	Fisher Exact/EASE score
**1** (0.72)	Transcription regulation	10 (14.9)	8.50E-05
	Neuron differentiation	11 (16.4)	1.90E-04
	Gamete generation	13 (19.4)	1.28E-03
	GTPase regulator activity	6 (9.0)	1.59E-03
	Metal ion binding	20 (29.9)	3.73E-03
	Microtubule cytoskeleton	7 (10.4)	2.92E-03
	Negative regulation of gene expression	7 (10.4)	5.13E-03
	ATP-binding	11 (16.4)	4.14E-04
	Microtubule motor activity	4 (6.0)	4.63E-03
	Ubl conjugation pathway	4 (6.0)	1.14E-02
	Ubiquitin-protein ligase activity	4 (6.0)	1.37E-02
	Regulation of developmental growth	3 (4.5)	2.61E-02
	Vision	3 (4.5)	2.83E-02
	Positive regulation of macromolecule biosynthetic process	3 (6.0)	4.73E-02
**2** (0.73)	Adenyl nucleotide binding	4 (40.0)	9.17E-03
	Microtubule cytoskeleton	3 (30.0)	1.19E-02
**3** (0.84)	No terms clustered		
**4** (0.68)	Cation channel activity	5 (35.7)	5.58E-06
	Integral to plasma membrane	5 (35.7)	3.04E-04
	Ion channel complex	4 (28.6)	9.71E-05
	Voltage-gated channel	3 (21.4)	9.65E-05
	Transmembrane	6 (42.9)	2.43E-03

Significant gene categories for each cluster were identified using DAVID (Pearson correlation coefficient shown in parenthesis next to cluster number). The top term in each cluster with a Fisher Exact/EASE Score p-value<0.05 are listed in this summary. The ‘Count’ column displays number of genes in each cluster associated with a particular GO term, percentage of the total number of genes in the cluster is shown in parenthesis.

### Functional testing of candidate genes

To validate the efficiency of this approach in identifying genes involved in the development of tolerance to sedation, we chose a sample of 19 genes to test by mutant and RNAi knockdown analysis. Gene-Expression Cluster #4 contained the *slo* gene, which has previously been shown to play a role in the production of ethanol tolerance. Based on the hypothesis that the Gene-Expression Clusters represent co-regulated genes and were therefore more likely to be involved in the same process, we focused our analysis on this cluster. In addition, Cluster #4 is highly enriched for genes associated with ion channel activity and synaptic membrane proteins and thus has the potential of modulating neural activity in response to drugs. Eleven candidates (out of thirteen cluster members) were selected from Gene-Expression Cluster #4 for mutant analysis. We also examined eight other candidates (five from Gene-Expression Cluster #1, one from Gene-Expression Cluster #2, and two that did not fall within any of the highly correlated clusters). The genes sampled were not chosen randomly but were selected based on the ease with which genetic tools could be obtained to test their function, whether or not the identity of the encoded protein was known, whether the gene was expressed in the nervous system, and prior information concerning the function of the gene (e.g. *pum* is a known activity-dependent regulator of neural activity [19]). This collection of genes consisted of four ion channel genes, three ion channel accessory genes, five transcription modulator genes, two protein kinase genes, one phosphatase gene, two cell adhesion genes, and two genes associated with cellular house-keeping (Table 2).

**Table 2:** Genes tested for drug tolerance using mutant analysis.

#	Gene Symbol	Molecular function	Flybase allele name tested	Allele class	Effect on tolerance	
					EtOH	BA
1	*Ack-like*	Cdc42-like tyrosine kinase	*Ack-like* ^KK105138^	*Tub*-Gal4: RNAi	**−**	**−**
1	*trr*	Histone methyl-transferase activity	*trr* ^GD4501^	*elav*-Gal4: RNAi	**−**	**−**
1	*msn*	Protein serine/threonine kinase	*msn* ^KK108948^	*elav*-Gal4: RNAi	**−**	**+**
1	*pum*	Translational repressor activity	*pum* ^13^	loss of function/hypomorphic	**+**	**+**
1	*nej*	Histone acetyl-transferase activity	*nej* ^3^/FM7	loss of function/heterozygous null	**+**	**+**
2	*unc-104*	Microtubule motor activity	*unc-104* ^HM05162^	*elav*-Gal4: RNAi	**−**	**−**
4	*nAcRα-30D*	Nicotinic acetylcholine receptor, α-subunit	*nAcRα-30D* ^DAS1^	loss of function/null	**−**	**−**
4	*eag*	Voltage-gated K^+^ channel, α-subunit	*eag* ^1^	loss of function/hypomorphic	**+**	**+**
4	*Teh2*	Voltage-gated Na+ channel, β-subunit	*Teh2* ^KK112449^	*Tub*-Gal4: RNAi	**+**	**+**
4	*slo*	BK-type Ca^2+^-activated K^+^ channel, α-subunit	*slo* ^4^	loss of function/null	**+**	**+**
4	*Ptp99A*	Protein tyrosine phosphatase	*Ptp99A* ^JF01858^	*Tub*-Gal4: RNAi	**+**	**+**
4	*mam*	Transcription co-activator	*mam* ^JF02881^	*elav*-Gal4: RNAi	**+**	**+**
4	*brp*	Ca^2+^ channel modulator activity	*brp* ^JF01932^	*elav*-Gal4: RNAi	**+**	**+**
4	*Ten-a*	Synaptic target recognition	*Ten-a* ^JF03375^	*Tub*-Gal4: RNAi	**+**	**+**
4	*Dscam*	Cell-surface immunoglobulin	*Dscam* ^JF03307^	*elav*-Gal4: RNAi	**−**	**−**
4	*kn*	Transcription factor	*Kn* ^JF02206^	*Tub*-Gal4: RNAi	**+**	**+**
4	*para*	Voltage-gated Na^+^ channel	*Para* ^JF01469^	*elav*-Gal4: RNAi	L	L
5	*so*	Transcription factor	*so* ^1^	loss of function	**−**	**−**
7	Act57B	Actin filament subunit	*Act57B* ^GD6854^	*Tub*-Gal4: RNAi	**−**	**−**

(#) Cluster number. (+) Significantly blocked or reduced tolerance. (−) No effect on tolerance. (L) Lethal.

To test the role of the candidate genes in alcohol tolerance, we used different gene knock-out/knockdown approaches. When possible, we used available loss-of-function null mutations, but in cases where the homozygous null mutations compromised viability of the stock, we used either hypomorphic alleles, null heterozygotes, or a transgene that carries a Gal4-inducible RNAi of the gene. Expression of the RNAi transgene was induced by crossing the RNAi stocks with either the ubiquitous *αTub84B*-Gal4 driver or, if this combination proved lethal, the more restricted pan-neural *elav-Gal4* driver was used. These distinct methods perturb gene expression in different ways and amounts, thereby increasing the chances of obtaining a viable adult. For the nineteen candidate genes, we used loss-of-function null alleles in three cases (*slo* ^4^, *so* ^1^, *nAChRa-30D* ^DAS1^), hypomorphic alleles in two cases (*pum* ^13^, *eag* ^1^), and a heterozygous loss-of-function allele in one case (*nej* ^3^/FM7). In addition, we used ubiquitously expressed RNAi alleles for six genes (*Act57B*, *Ack-like*, *Teh2*, *Ptp99A*, *Ten-a*, *kn*) and neurally restricted RNAi alleles for another six (*brp*, *msn*, *trr*, *unc-104, mam, Dscam*). Only one gene candidate was lethal under both RNAi induction protocols and thus could not be behaviorally tested (*para*). For the purpose of convenience, in the remainder of the manuscript the word *mutant* will refer to all of the allele types used.

**Figure 3:**
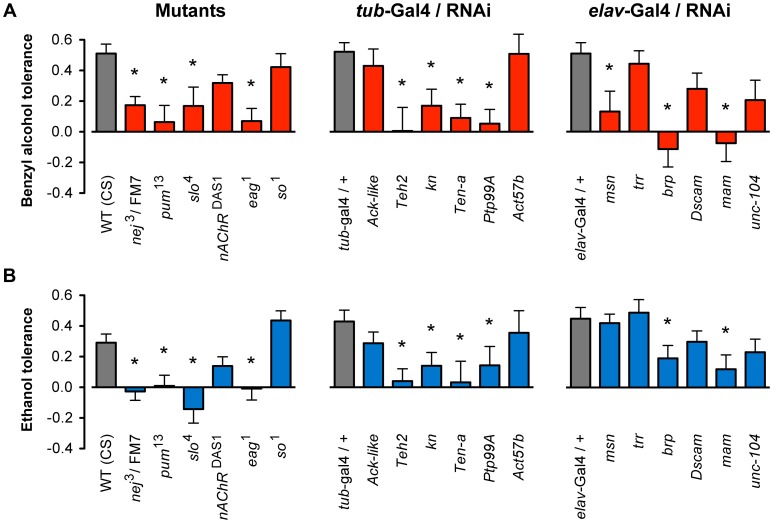
Mutant and RNAi behavioral analysis of benzyl alcohol and ethanol tolerance. **A**) Mutant alleles or inducible RNAi lines for eighteen genes were tested for the ability to acquire tolerance to benzyl alcohol. Eleven of these lines significantly block or reduced benzyl alcohol tolerance as compared to their appropriate controls. **B**) Mutant alleles or inducible RNAi lines for the same eighteen genes shown in (**A**) were tested for the ability to acquire tolerance to ethanol. Ten of these lines significantly block or reduced tolerance as compared to their appropriate controls. Controls were: wild-type (WT) CS strain for mutants, and the respective heterozygous Gal4-driver transgenic for RNAi lines. * denotes significant difference in magnitude of tolerance between subjected lines and controls (P<0.05).

For each mutant stock, the animals were subjected to a two-day alcohol tolerance assay. In this assay, a population of female flies was divided into two groups. On the first day, one group (experimental) was sedated with either benzyl alcohol or ethanol vapor, whereas the second group was left untreated (control). On the second day, both groups were sedated with the alcohol vapor and the time of recovery monitored. If the experimental group recovered faster than the control group, the strain was said to be capable of acquiring tolerance. The magnitude of tolerance (i.e. the change in recovery time between experimental and control groups) was determined for each strain and statistically compared to the appropriate wild-type controls. The magnitude of benzyl alcohol tolerance for each strain is plotted in Figure 3A. Mutations in eleven of the eighteen genes tested significantly blocked or reduced tolerance to benzyl alcohol, while for the remaining seven genes, the mutation did not significantly affect benzyl alcohol tolerance. To determine if these mutations disrupt ethanol tolerance, all alleles were subjected to an equivalent two-day ethanol tolerance assay. Of the eleven alleles that significantly disrupted benzyl alcohol tolerance, ten also disrupted ethanol tolerance, and the remaining eight did not affect ethanol tolerance (Figure 3B). These results reflect a validation rate of approximately 55% success for alcohol tolerance. Most importantly within Cluster #4, the success rate is even higher —80% behavioral validation. Moreover, only one of the genes tested affected tolerance to one of the drugs but not both (the mutation in *msn* significantly reduced benzyl alcohol tolerance but failed to affect ethanol tolerance significantly). Together, these data confirm our central hypothesis, that similarities in the histone modification profiles between benzyl alcohol- and ethanol-sedated flies is a useful way to identify genes that are involved in the acquisition of functional tolerance.

During mutant analysis, we confirmed that each iteration of the treatment protocol produced tolerance by performing the tolerance test with the Canton-S (CS) strain—a common wild-type control used in the community. For each of the RNAi knockdown experiments, we used the progeny of the cross between the respective Gal4-driver strain and the *w* ^1118^ strain as genetic controls. The *w* ^1118^ strain carries the same genetic background as all the RNAi lines tested and therefore represents an appropriate genetic control for the progeny of the induced lines. The RNAi transgenic inserts are carried in two different chromosomal sites. The insertion site of the RNAi transgenes obtained from the TRiP Transgenic RNAi Project are all inserted into an engineered attP2 site at position 3L:11,063,638 (chromosome 3), while the insertion site of the RNAi transgene in lines obtained from the Vienna Drosophila RNAi Center are inserted at position 2L:22,019,296 (chromosome 2). It is possible that insertions at these positions disrupt expression of a gene important for tolerance. This has been ruled out by the fact that not all of the RNAi inserts at these position affect tolerance. Two additional RNAi lines had inserts at unmapped locations; however, neither of these affected tolerance.

### Gene-expression analysis of candidate genes

Changes in histone acetylation are known to be associated with changes in transcriptional activity of genes. Because the genes validated here were first identified through alcohol-induced changes in histone acetylation, one would expect that the transcriptional activity of these genes would also be modulated by alcohol exposure. To examine if these genes display alcohol-induced changes in gene expression, we performed quantitative RT-PCR analysis of mRNA abundance for a set of the candidate genes in response to either ethanol or benzyl alcohol. The genes tested here include *eag*, *brp*, *Teh2*, *pum*, *nej*, and *para*.

**Figure 4:**
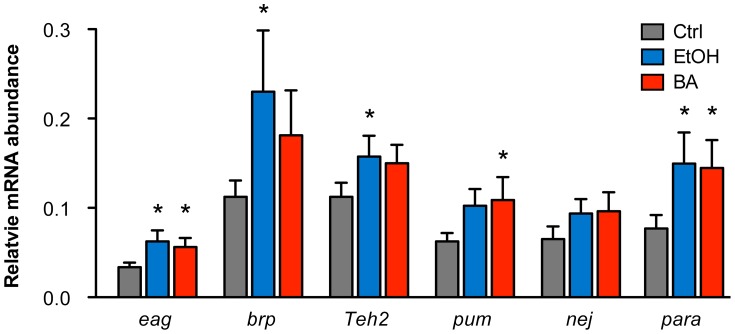
Alcohol-induced upregulation of expression of candidate genes. The relative mRNA levels for candidate genes *eag*, *brp*, *Teh2*, *pum*, *nej*, and *para* in control animals, benzyl alcohol-sedated animals, and ethanol-sedated animals are shown. Abundance of mRNA for each gene was determined by quantitative reverse-transcription PCR analysis and expressed relative to the abundance of the *Cyp1* gene. Error bars are SEM. Asterisks denote statistically significant differences from the untreated controls (P<0.05, One-way ANOVA with Dunnett's post-hoc test). Overall statistical significance of the effects of alcohol treatment for the whole set of genes was determined by Two-way ANOVA (P = 0.013).

As shown in Figure 4, we confirmed that all candidate genes tested increase mRNA expression in response to both ethanol and benzyl alcohol. While there are a few individual instances in which the message upregulation does not reach statistical significance, the overall effect of alcohol treatment is statistically significant amongst the six genes (P = 0.0013, by Two-way ANOVA). For individual cases, the genes *eag* and *para* showed significant upregulation after both ethanol and benzyl alcohol. The genes *brp* and *Teh2* show a significant upregulation only after ethanol treatment, while *pum* showed upregulation only after benzyl alcohol treatment. Previous studies have shown that *slo* is slightly induced by both benzyl alcohol and ethanol sedation [10], [12]. The *nej* gene may be upregulated by both drugs, albeit the changes reported here did not reach statistical significance. However, this might be a consequence of assaying for changes in gene expression at only the 6 h post-sedation time point. We expect that a time course analysis following alcohol exposure will be required to authoritatively assess the transcriptional dynamics of each gene's response to alcohol sedation.

## Discussion

Because many histone modifications are a direct molecular consequence of transcription factor/co-factor activity, they represent a reliable indicator of alterations in gene activity [20]. Patterns of histone modifications can be used to identify genes that are coordinately regulated and to identify the position of enhancers that mediate gene induction [21], [22]. The histone H4 acetylation changes produced by benzyl alcohol and ethanol are grossly different, even though these drugs generate mutual cross-tolerance that has overlapping molecular origins [12]. However, we reasoned that the intersection between the benzyl alcohol pattern and the ethanol would be enriched for genes that underlie common adaptive tolerance response. Because, alcohol tolerance and dependence are thought to arise from common homeostatic mechanisms [23], we believe that some (but not all) of the genes identified here are also likely to have a role in the development of alcohol dependence.

We identified a cohort of 144 genes whose histone H4 acetylation state increases with either drug. To further organize the candidates into functionally-related groups we performed cluster analysis based on the transcriptional responses to a variety of different treatments. Because the expression of each subgroup is highly correlated, the genes in a group are likely to be coordinately regulated. This analysis was combined with Gene Ontology Term Clustering to generate subgroups enriched for genes that share both common transcriptional responses and participate in common biological processes. Of the eighteen genes tested for tolerance, ten were validated by mutant analysis. Although, mutant analysis in flies is faster than in mammals, the tolerance assay is sufficiently time consuming to make an unbiased genetic screen unattractive. However, this analysis shows that prescreening with this method reduces the number of candidates to be tested to a manageable number. We expect that analysis of the remaining 125 genes will continue to identify new tolerance genes, although as we move deeper into the rank-ordered list the success rate may decline.

The genes implicated in producing ethanol tolerance have striking interrelationships linked to specific cellular processes. All ten validated genes have previously been associated with the regulation of neural physiology, neural development or synaptic plasticity, implicating a role for coordinate regulation of neural activity as a means to achieve long-term adaptations to alcohol. While it is intuitively obvious that functional tolerance to these drugs would involve adaptations affecting neural activity, our screen implicates specific genes. Historically, Drosophila has been an excellent model system for describing the cascades of interacting genes [14] that underlie a specific response. However, the first step in this process is the identification of a collection of mutations that specifically affect the response being studied. We believe that we have described an excellent method for enriching for tolerance genes.

One gene identified by our unbiased enrichment procedure is the *slowpoke* (*slo*) BK-type K^+^ channel gene. This represents a key validating result because this gene has previously been shown to be important for ethanol and benzyl alcohol tolerance in Drosophila [2], [12] and has also been shown to play a central role in the development of alcohol tolerance in *C. elegans* and mammals [24], [25]. The *slo* gene encodes the pore forming subunit of the BK-type Ca^2+^-activated K^+^ channel, a pre-synaptic channel directly involved in the regulation of action potential shape and neurotransmitter release [26]–[28]. In Drosophila, an alcohol-induced increase in *slo* expression has been shown to enhance neural capacity for repetitive firing, resulting in enhanced resistance to the sedative effects of alcohol (tolerance), and increased susceptibility to alcohol withdrawal seizures—a symptom of alcohol dependence [14], [15]. The mammalian homolog of *slo* is KCNMA1 [29].

A second gene validated as being required for functional tolerance is the *eag* K^+^ channel gene. This is intriguing because the *eag* gene genetically interacts with the *slo* gene. In Drosophila, *eag* has been proposed to contribute subunits to ion channels ostensibly considered to be products of other ion channel genes, including BK channels [30], [31]. This claim is buttressed by the recent finding that, in *C. elegans*, the *eag* and *slo* homologs genetically interact [32]. On a related note, heterologous expression studies in mammalian cells show evidence that abused drugs such as ethanol and cocaine significantly block channels produced by the human *eag* homolog [33], [34]. The mammalian homologs of *eag* are members of the ERG/KCNH family of delayed rectifier voltage gated K^+^ channel genes [35].

The *pum* and *Teh2* genes encode proteins that interact with the *para* voltage-gated Na^+^ channel, which also appears in our list. The gene *pumilio* (*pum*) encodes an RNA-binding protein that regulates translation and mRNA stability by binding to the 3′-UTR of mRNAs [36]–[38]. In addition to roles in germline development and embryogenesis, *pum* has been directly linked to the activity dependent regulation of neuronal excitability, pre-synaptic morphology, and long-term memory [19], [39], [40]. In neurons, the Pum protein regulates the translation of *para* mRNA in an activity-dependent manner [41] and therefore is a prime candidate for modulation of ethanol response that contributes to tolerance. Furthermore, a previous mutant screen had identified *pum* among a collection of learning and memory mutants as being involved in the development of rapid tolerance to alcohol [42].

The *Teh2* gene encodes a member of a structurally conserved family of ion channel β-subunits. Teh2 has been functionally shown to act as β-subunit of the Para voltage-gated Na^+^ channel whose presence alters channel activity [43]. We have not yet properly evaluated whether the mutations in *para* itself would interfere with tolerance. The temperature sensitive *para* ^ts1^ allele does not block tolerance at permissive temperatures [44], [45], but animals carrying this temperature-sensitive mutation are essentially normal at the permissive temperature and completely paralyzed at the restrictive temperature. Unfortunately, induction of *para* RNAi with both Gal4 drivers used in this study resulted in lethality (*para* is an essential gene). Nevertheless, the identification of both *Teh2* (a Para voltage-gated Na^+^ channel auxiliary subunit) and *pum* (a translational repressor of *para* mRNA) strongly implicate Para Na^+^ channels as playing an important role in alcohol tolerance. This hypothesis can eventually be tested by collecting and testing hypomorphic *para* alleles.

Surprisingly, the Teh2 protein also shows strong topological homology to the human *slo* BK channel β subunit, and it has been postulated (but not yet proven) that it could also act as a β-subunit for *slo* BK channels in flies [43]. The expression profile of *Teh2* is very similar to the expression profile of *slo* [46], suggesting that both proteins are expressed in the same cells. In mammals, both Na_V_ and BK channel β-subunits serve as key regulators of their respective α subunits [47], [48]. Furthermore in mammals, modulation of BK channels by β-subunits plays a role in the regulation of the molecular and behavioral responses to alcohol [49], [50]. Further work is required to determine whether Drosophila Teh2 and BK channels interact.

The gene *brp* encodes a pre-synaptic active zone component with significant sequence homology to a neural isoform of the vertebrate ELKS/CAST/ERC family. The Brp protein has been shown to be a critical player in the assembly of active zones and the regulation of evoked neurotransmitter release at chemical synapses [51]. Because of its physical interaction with pre-synaptic Ca^2+^ channels, Brp is thought to play an important role in clustering Ca^2+^ channels and vesicles to allow efficient transmitter release and synaptic plasticity [52], [53]. Although there are no previous reports of interactions with drugs of abuse, *brp* is a key candidate for the control of synaptic homeostasis at the pre-synaptic active zone [54], [55].

A number of genes identified in this study encode proteins involved in synaptic growth and axon guidance. For instance, the gene *Ptp99A*, a transmembrane receptor protein tyrosine phosphatase [56], is involved in motor neuron axon guidance [57] and defasciculation of motor neuron axon [58]. The gene *Ten-a* encodes a protein with unknown function. However mutant phenotypes indicate it is important in synaptic target recognition, attraction and growth [59]–[61]. Meanwhile, the gene *knot/collier* (*kn*/*col*) is a sequence-specific DNA binding transcription factor involved in dendrite morphogenesis [62] and plays roles in the innate immune response [63]. Interestingly, the innate immune response has recently been linked with alcohol phenotypes in both flies and mammals [64], [65]. However, the mechanisms by which immune factors contribute to behavioral changes associated with alcohol exposure remain unclear. Based on the gene-expression similarities with the myriad of synaptic genes found here, the gene *kn* could link the neuroimmune response to alcohol-induced behavioral changes.

In addition to synaptic and neurogenic factors, two other transcription modulators were identified. The gene *nej*, which encodes the Drosophila homolog of the mammalian transcriptional co-activator CREB-binding protein (CBP) [66], was also found in this study. CBP is recruited to DNA sites by a number of transcription factors, including CREB and cFos, and acts as a histone acetyl-transferase (HAT), and thus, it is associated with activation of gene expression [67]–[69]. While most studies of Drosophila CBP have focused on its role during development [69]–[71], a significant contribution of CBP to the regulation of pre-synaptic function has also been reported [72]. Furthermore, several lines of evidence indicate that through its interactions with CREB, CBP plays a critical role in the activity-dependent regulation of neural excitability and synaptic plasticity [73], [74]. In Drosophila, CREB has already been shown to be involved in producing tolerance through the regulation of *slo* transcription [13], and in this role CREB probably employs CBP. In mammals, CBP has been shown to modulate both ethanol and cocaine associated behaviors through the acetylation of histones [6], [75], [76].

Finally, the gene *mastermind* (*mam*) is a transcription factor co-activator that has been involved in nervous system development [77]–[79]. In mammals, Mam has been shown to directly associate with the histone acetyl transferase CBP/p300 with which it mediates chromatin-specific transcription. Furthermore, Mam induces phosphorylation and localization of CBP/p300 proteins to nuclear foci [80]. We have now implicated both *mam* and *nej* (which encodes CBP) in mediating tolerance to alcohol and believe that this same transcriptional regulating complex may be a central regulator of other neuroadaptations to alcohol.

Ion channels and synaptic proteins work together to fine-tune cell excitability and synaptic communication. It is expected that environmental insults that affect neural activity will precipitate compensatory mechanisms that homeostatically regulate neural excitability. This may involve the coordinate modulation of a network of genes. Elucidating the networks of proteins that work together in regulating neural adaptation to alcohol provides a powerful way to understand the integrative mechanisms that lead to addiction. Here we have come a step closer by identifying a small network of neural genes with the potential of regulating neural activity in the development of an addiction phenotype: tolerance.

**Figure 5:**
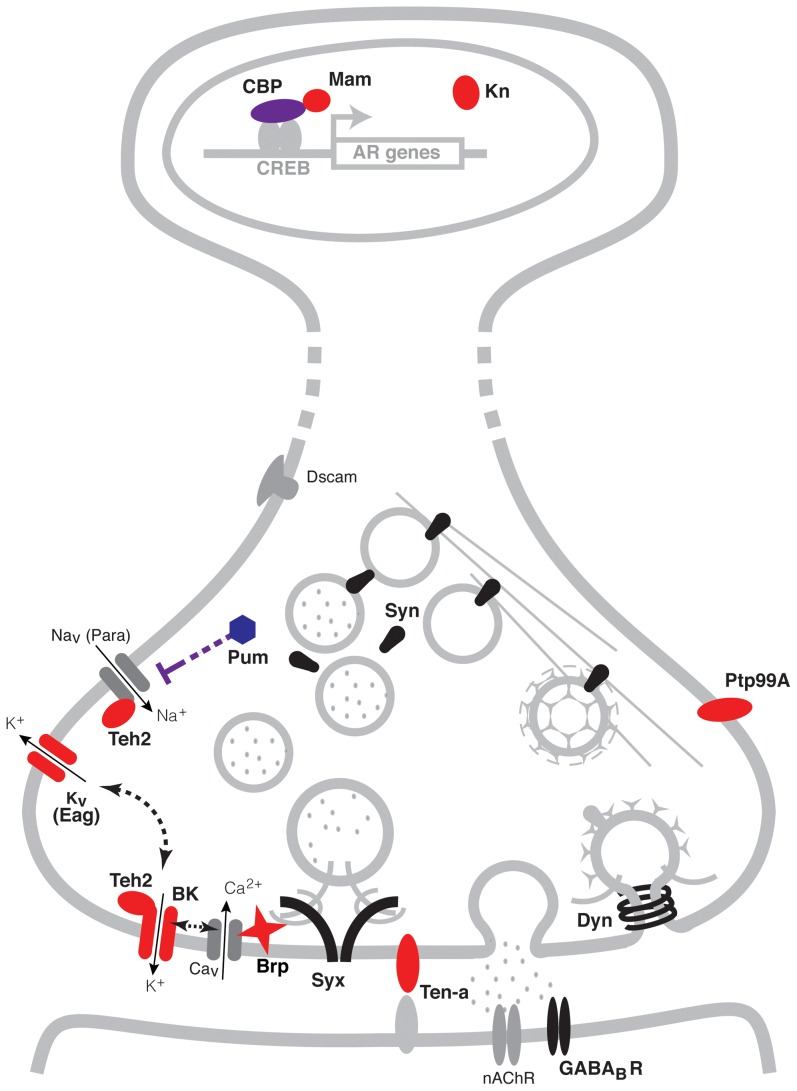
A pre-synaptic network model of alcohol tolerance. Schematic representation of the cellular location of the proteins encoded by the cohort of genes identified in this study. Proteins depicted in blue belong to expression cluster #1, while those in red belong to cluster #4. Seven of the ten genes identified encode proteins that reside at the pre-synaptic terminals of neurons. Two encode ion channel proteins directly involved in the modulation of neural excitability (Eag and BK), two encode proteins that possess ion-channel regulator roles (Pum and Teh2), one encodes a pre-synaptic active zone component that provides support to transmitter release (Brp) and two encode transmembrane proteins involved in neuronal morphogenesis (Ptp99A and Ten-a). Three other genes positively identified to affect tolerance encode transcription modulator proteins. These are the CBP histone acetyl-transferase encoded by *nej*, the transcription co-activator Mam and the innate immune factor Kn. Other synaptic proteins previously associated with alcohol tolerance, are depicted in black. These proteins include Synapsins (Syn), Dynamin (Dyn), Syntaxin 1A (Syx) and the GABA_B_ post-synaptic receptor (GABA_B_R). While all these genes have known roles in specific synaptic processes, together with the genes identified here, they have the capacity to orchestrate neural adaptation in response to alcohol. Proteins and structures in gray are included to provide context.

As a summary, Figure 5 shows a schematic representation of the genes identified here within a representative neuron. Possible regulatory interactions between many of these genes become immediately apparent. At the transcriptional level for instance, CBP (encoded by the gene *nej*) has a direct role in histone acetylation of chromosomal regions through its interactions with the Transcription factor CREB [67]. The CBP/CREB assembly has being extensively involved in the transcriptional regulation of gene targets and is particularly associated with the activity-dependent regulation of synaptic plasticity [72], [73]. Furthermore, the transcription co-activator Mam, which associates with CBP, may serve as a modulator of the transcriptional response of target genes [80]. At the translational level, a direct interaction between the translational repressor Pum and the Para voltage-gated Na^+^ channel has also been reported in neurons [41]. Meanwhile at the protein level, many known interactions also exist. For example, the putative ion-channel β subunit Teh2 is known to modulate the activity the *para* voltage-gated Na^+^ channel, and has also been postulated to interact with the BK channel encoded by *slo* [43]. In turn, BK channels can modulate the activity of voltage-gated K^+^ channels such as Eag and vise-versa [30], [81], as well as the release of neurotransmitter through its interactions with voltage-gated Ca^2+^ channels. The active zone component Brp is also thought to play an important role in the regulation of transmitter release through its interactions with Ca^2+^ channels and the synaptic vesicles complex [52], [53]. Altogether, coordinately increasing the expression of all of these proteins could have a strong effect on synaptic activity.

A previous genome-wide gene-expression study in flies showed that two hours after ethanol exposure there are changes in the expression of a suite of genes encoding chemosensory receptors, detoxification enzymes, and metabolic enzymes [82]. Changes in the expression of these genes may represent sensory and metabolic adaptation to ethanol. However, only few of these genes were tested for a role in functional tolerance and examining each of the genes identified by mutant analysis would be a lengthy endeavor. Instead, the same group surveyed a large collection of wild-derived inbred lines of Drosophila for differences in gene expression that correlated with the magnitude of tolerance induced by a single ethanol exposure [83]. In this study, the authors linked a module (cluster) of synaptic genes to the capacity to generate tolerance. The module included *Synapsin, comt* (a gene that encodes the NSF protein that mediates ATP-dependent synaptic vesicle release), the soluble NSF attachment protein gene *Snap*, and the SNAP receptor genes *Snap25* and *Syx16* (amongst others). Single gene mutant analysis has identified several additional synaptic genes as being required for alcohol tolerance. These genes encode the pre-synaptic proteins Dynamin, Syntaxin 1A, and Synapsin [44], [45], [84]; the transmembrane cell adhesion integrin subunit βPS and αPS3 [85]; the postsynaptic GABA_B_ receptor [86]; and the post-synaptic scaffolding protein Homer [87]. However, none of these genes appear in our list of genes that show similar benzyl alcohol and ethanol-induced histone acetylation patterns.

While there is very little overlap of our candidate genes with those described above, there is a strong overlap in biological function. One possible reason for the lack of overlap is that the distinct methodologies used offer unique glimpses into the mechanism of tolerance. For instance, mRNA abundance can be altered by regulated control of mRNA stability and protein activity can also be regulated post transcriptionally–both of which would not be visible in a ChIP-chip assay. The ChIP-chip assay should visualize changes in chromatin preferentially associated with transcription. On the other hand, the inbred line approach might best work for identifying genes that alter the predisposition for alcohol tolerance but may not flag those genes that change expression in order to implement the tolerance response. We are searching for genes that mediate the plastic changes that implement tolerance. We believe that the coincidence between benzyl alcohol- and ethanol-induced histone acetylation acts as a filter that helps enrich for tolerance genes by removing genomic responses irrelevant to the shared behavioral effects of the drugs. Combining this with co-expression network analysis and gene ontology clustering results in a highly effective enrichment procedure. We are convinced that together with the genes identified in previous studies, the genes identified here will help complete the puzzle of a very complex response.

There is strong conservation of gene regulatory networks between Drosophila and mammals and a remarkable evolutionary concordance in the genes that underlie drug tolerance [88], [89]. The gene networks identified here will be immediately useful for the identification of genes and regulatory events important for tolerance, dependence, or addiction in mammals. Drosophila still has an important role to play in that it is an ideal model organism for deciphering how this large collection of genes interact to produce an ethanol-induced response. The effectiveness of the technique used here relies primarily on the combinatorial approach, as the genomic-level ChIP data was of relatively low power (N = 2/group) and yet the combined approach was remarkably successful. This approach may be useful for characterizing other types of complex polygenic responses.

## Materials and Methods

### Fly stocks

Drosophila stocks were raised on standard cornmeal agar medium in a 12/12 h light/dark cycle. For all assays, newly eclosed flies were collected over a two-day interval and studied 3 to 5 days after collection. The wild-type stock Canton S (CS); the mutants stocks *eag* ^1^, *nej* ^3^/FM7, *pum* ^13^, *so* ^1^, *nAcRα-30D* ^DAS1^; the RNAi lines *brp* ^JF01932^, *para* ^JF01469^, *unc-104* ^HM05162^, *Kn* ^JF02206^, *Ten-a* ^JF03375^, *Ptp99A* ^JF01858^, *Dscam* ^JF03307^, *mam* ^JF02881^; and the Gal4 drivers *elav*[C155]-Gal4;UAS-*Dcr2* and *tubP*-Gal4; were all obtained from the Bloomington Drosophila Stock Center at Indiana University (Bloomington, IN). *The UAS-RNAi lines Act57B* ^GD6854^, *trr* ^GD4501^, *Teh2* ^KK112449^, *Ack-like* ^KK105138^, *msn* ^KK108948^ and the isogenic host strain for the RNAi library *w* ^1118^, were obtained from the Vienna Drosophila RNAi Center (VDRC) [90]. For RNAi induction, each RNAi lines was crossed to the *tubP*-Gal4 line or the *elav*[C155]-Gal4;UAS-*Dcr2*, and the progeny tested. The wild type stocks used for comparisons were CS for all the mutant lines, and the progeny of the cross between *w* ^1118^ and the respective Gal4 driver line for the RNAi stocks. The *slo* ^4^ mutant was obtained from the Atkinson Lab collection.

### Drug treatments

Approximately 500 age-matched wild-type CS flies were collected for exposure to either ethanol, benzyl alcohol, or for use as the respective untreated controls. For benzyl alcohol, the insides of a 180 ml glass tube were coated with 500 ul of a 0.4% benzyl alcohol solution in acetone. The tube was continuously rotated for 30 minutes at room temperature to evaporate the acetone, leaving a thin coat of evenly distributed benzyl alcohol. For the untreated control, a similar acetone-only tube was prepared. Flies were placed in each tube and exposed until the benzyl alcohol group was completely sedated (approximately 15 minutes) [10]. Flies were then transferred to fresh-food bottles for recovery. For ethanol exposure, flies were placed in a perforated 500 ml plastic bottle chamber. Humidified air saturated with ethanol vapor was delivered to flies in the chamber using an ethanol vapor inebriator set to 15 ml air per minute. For the untreated control, ethanol free humidified air was delivered to the chamber. Flies were placed in each chamber and exposed until the ethanol group was completely sedated (approximately 15 minutes) [11]. Flies were then transferred to fresh-food bottles for recovery.

### Chromatin immunoprecipitation and genome-wide tilling arrays analysis (ChIP-chip)

Chromatin extraction and immunoprecipitation were performed as described previously by [5]. In brief, formaldehyde cross-linked chromatin was extracted from 500 fly heads of drug treated and control flies (mix of males and females), 6 hours after treatment and fragmented by sonication to ∼500 bp length. Chromatin samples (2 ug) were immunoprecipitated using a 1∶200 dilution of ChIP grade antibody against acetylated histone H4 at lysine 5, 8, 12 and 16 (catalog # 06-866; EMD Millipore, Billerica, MA). A fraction (1/10) of the chromatin sample was left unprecipitated for use as input control. DNA from immunoprecipitated (ChIP) and input samples was washed, reversed cross-linked, and purified and subsequently amplified using the GenomePlex Complete Whole Genome Amplification Kit (Sigma-Aldrich, St. Louis, MO) following the manufacturer's protocol. Approximately 1 ug of amplified ChIP and input DNA from each sample was sent to NimbleGen (Roche NimbleGen, Madison, WI) for two-color hybridization to Drosophila ChIP-chip 2.1M Whole-Genome Tiling Arrays. Each array consisted of 2.1 million 50–75 mer probes, with a 55 bp median probe spacing that cover the entire Drosophila genome (UCSC Drosophila genome build DM3). For measurements of control baseline H4 acetylation profiles, control ChIP and input DNA samples were labeled with Cy3 and Cy5 fluorescent dye, respectively, and co-hybridized to the same microarray. ChIP acetylation signals were reported as normalized Log2 ChIP_(control)_/Input_(control)_ ratios. For measurements of drug-induced changes in acetylation, the control and drug-treated ChIP DNA samples were labeled with Cy3 and Cy5 fluorescent dye, respectively, and co-hybridized to the same microarray. The difference in acetylation signals were reported as normalized log2 ChIP_(drug-treated)_/ChIP_(control)_ ratios. Each ChIP-chip experiment was repeated two times from independent biological samples. For each experiment, raw signals of corresponding experimental replicates were normalized using the ‘vsn’ package for R [91] and signal ratios from replicates were averaged using R/Bioconductor (www.R-project.org; www.bioconductor.org) [92], [93] according to an online protocol (http://epigenesys.eu/images/stories/protocols/pdf/20111025114444_p43.pdf). Only arrays with normally distributed log-transformed signals were used, and signal normalization between arrays was performed against matched samples. Signal ratio peaks with enrichment score above 50% and a false discovery rate (FDR) of <0.05 and mapped to the annotated Drosophila genome (UCSC, build DM3) using NimbleGen SignalMap software following default parameters. Peaks were assigned to the nearest gene using a bidirectional distance cut off of 500 bp beyond the annotated gene region defined by the 5′-most transcriptional start site to the end of the 3′ UTR. This analysis produced approximately 1500 associated genes for each drug. Genes were rank ordered with respect to peak magnitude. The genes mapped to peaks produced by benzyl alcohol and ethanol treatment were compared for common entries using Microsoft Excel for Mac software (Microsoft, Redmond, WA).

The raw and processed data from the ChIP-chip data described in this manuscript have been deposited in the public functional genomics data repository from NCBI: Gene Expression Omnibus (GEO). Data can be found on the GEO website (http://www.ncbi.nlm.nih.gov/geo/) using accession number GSE48449. All essential sample annotation and experimental design information including sample data relationships have been included in the repository according to the Minimum Information About a Microarray Experiment (MIAME) guidelines [94].

### Tolerance assays

For all tolerance assays, 5 to 7 day old age-matched female offspring from each line tested were collected and sorted into replicate vials under light CO_2_ anesthesia. To test for benzyl alcohol tolerance, flies were divided into 2 equal groups: the control group and the experimental group. Each group consisted of three vials with 12 flies each. On the first day, flies from each vial of the experimental group were sedated using a custom build benzyl alcohol vapor chamber for 15 minute, while the control group was mock sedated [14]. After sedation, the animals were returned to food vials for 24 hours allowed to recover. On the second day, both groups were sedated in tandem using the same benzyl alcohol vapor chambers. This time, flies were transferred on to small plastic Petri dishes immediately after sedation and their recovery period monitored. Flies were said to have recovered from sedation once they regain postural control. Sedation recovery was quantified by counting the number of flies recovered from sedation in each vial at 5-minute intervals. Recovery scores for each vial were plotted as the percentage of flies recovered from sedation over time. For ethanol, tolerance was assayed as previously described [45]. In brief, flies were divided into 2 groups of equal numbers: the control group and the experimental group. Each group consisted of six vials with 10 flies each. On the first day, the experimental group was sedated using an ethanol-saturated air stream, while the control group was mock sedated. After sedation, the animals were allowed to recover in a fresh air environment and then returned to food vials for 24 hours. On the second day, both groups were sedated in tandem using the same ethanol-saturated air stream method. Again, after sedation, the ethanol vapor was replaced with fresh air, and their recovery period monitored. Flies were said to have recovered from sedation once they regain postural control. Sedation recovery was quantified by counting the number of flies recovered from sedation in each vial at 2-minute intervals. Recovery scores for each vial were plotted as the percentage of flies recovered from sedation over time. For both benzyl alcohol and ethanol, tolerance was determined by comparing the 50% recovery time from sedation between the control and experimental groups. The 50% recovery time (and the associated SEM) for each treatment group was calculated by performing a Richard's five parameter non-linear regression curve fit on the respective recovery curves using GraphPad Prism for Mac software (GraphPad Software, La Jolla, CA). The relative change in recovery time (magnitude of tolerance) between the experimental and control groups was determined by calculating the difference in the 50% recovery time between the groups and compared to the magnitude of tolerance of the respective control lines. Statistical significance was determined using one-way ANOVA followed by Dunnett's multiple-comparison post hoc test.

### Pearson correlation and gene clustering analysis

Gene-expression data for the 144 genes identified in this study was obtained from the Drosophila database FlyBase [95]. This data was collected by the modENCODE project [16] from RNA-Seq analysis performed on poly(A)+ RNA from Drosophila treated with various chemicals through feeding or subjected to temperature shock. Total RNA isolation, poly(A)+ RNA purification and strand-specific library construction were performed in the Brenton Graveley and Peter Cherbas groups. Libraries were subjected to paired-end RNA sequencing (2×76+ nt) on the GAIIx and HiSeq platforms (Celniker, Gingeras, and Graveley groups). Fastq files were generated using pipeline version 1.5. Treatment conditions are listed in Supporting Table S1.

Gene clustering analysis based on treatment co-expression profiles was performed by the Cluster 3.0 for Mac OSX program [96]. For this, gene-expression data was prepared for Cluster by importing the 21 RNA-Seq expression datasets of the fly populations exposed to distinct external treatment conditions for the 144 genes identified from common acetylation changes. First, a self-organizing map (SOM) was made using default parameters (10 clusters) based on Pearson's correlation (centered) similarity matrix of each gene-expression profiles. The resulting SOM file was then used to perform complete-linkage hierarchical clustering of the normalized gene-expression profiles. For genes expression normalization Cluster 3.0 multiplies all values in each row of data to a scale factor S to so that the sum of the squares of the values is in each row is 1.0 (a separate S is computed for each row). Hierarchical clustering was applied to both rows and columns. For heat-map visualization, the output file was exported to JavaTreeview [97]. Pearson's Correlation coefficients of the gene-expression data from D. melanogaster exposed to the various conditions for each pair of the 13 tested genes was performed using GraphPad Prism for Mac software (GraphPad Software, La Jolla, CA). For gene ontology annotation search and clustering, significant gene categories for each cluster were identified using DAVID web-accessible version 6.7 [18] with default parameters (High, 3, 0.85, 3, 3, 0.5) and official gene symbols as input.

### Validation of ChIP-chip data by qPCR

To test the validity of the genome-wide arrays for accurately reporting significant acetylation peaks we used quantitative real-time PCR (qPCR) to measure representative acetylation signals (ChIP/Input) from independent chromatin samples. Primer sets were designed for 10 unique loci across the genome. These loci mapped to the promoter region of 8 different genes (*Creb2*, *CrebA*, *Cyc*, *dbi*, *gpdh*, *pdf*, *per* and *Rdl*). Primer sequences are displayed in Supporting Table S2.

Real-time PCR analysis of ChIP DNA was performed using the SYBR Green PCR Master Mix (Applied Biosystems/Life technologies, Carlsbad, CA) in an ABI Prism 7300 Sequence Detection System (Applied Biosystems, Carlsbad, CA) as described previously by [5]. ChIP/Input ratios reported for each primer set are averages and SEM of three chromatin samples (Supporting Figure S1A). For comparison, signal peaks of the same genomic loci were extracted from one of the NimbleGen ChIP-chip data sets. For this, ChIP/Inputs ratio signals from 7 consecutive probes spanning the center of the region defined by each primer set used in the qPCR experiment were grouped an the average and SEM signal calculated (Supporting Figure S1B). Correlation analysis of the signal ratio profile generated by the ChIP-chip and ChIP-qPCR data across the 10 unique regions was performed using Pearson's correlation coefficient analysis. The ChIP-qPCR and the genome-wide ChIP-chip signals display a very high correlation coefficient (r = 0.849, P value = 0.0019).

### Gene-expression analysis of candidate genes by qPCR

Total RNA was extracted from heads of groups of 100 flies (mix of males and females) 6 hours after treatment with either ethanol or benzyl alcohol, and from untreated controls, using a single-step RNA isolation protocol [98]. Residual genomic DNA was digested by incubating the RNA samples at 37°C for 30 min with RNase free DNase I (Ambion, Austin, TX) and further purified by acid phenol/chloroform extraction (Ambion, Austin, TX) and ethanol precipitation. RNA quality was determined by agarose gel electrophoresis and quantified using a NanoDrop Spectrophotometer (NanoDrop Technologies, Wilmington, DE). First-strand cDNA was synthesized from 50 ng of total RNA using the SuperScript VILO cDNA Synthesis Kit (Invitrogen/Life technologies, Carlsbad, CA). The cDNAs were amplified by real-time PCR using the SYBR Green PCR Master Mix (Applied Biosystems/Life technologies, Carlsbad, CA) in an ABI Prism 7300 Sequence Detection System (Applied Biosystems, Carlsbad, CA) following the manufacturer's protocols. Quantification of the starting mRNA for each gene was determined relative to the *Cyp1* mRNA using the ΔΔCt method. Primer sequences for the genes tested are displayed in Supporting Table S3. A total of 8 replicate RT-PCR reactions were performed from independent RNA samples, and the yields thereof were expressed as an average. Statistical significance was calculated using the One-way ANOVA for each gene with Dunnett's post-hoc test for comparisons to the untreated controls. Statistical significance for the effects of alcohol treatment on gene expression for the group of genes was determined by Two-way ANOVA.

## Supporting Information

Dataset S1 Complete annotated list of genes with increased acetylation after ethanol and benzyl alcohol exposure (Supporting_Dataset-S1.xls).
(XLS)

Dataset S2 Complete list of Gene Ontology Terms for each Gene-Expression Cluster (Supporting_Dataset-S2).
(XLS)

Figure S1 qPCR validation of NimbleGen ChIP-chip data. Shown are ChIP/Input ratios for 10 different gene loci as measured by qPCR or a NimbleGen DNA tilling array. **A**) Primer sets were designed for 10 unique loci across the genome mapping to the promoter region of 8 different genes (*Creb2*, *CrebA*, *Cyc*, *dbi*, *gpdh*, *pdf*, *per* and *Rdl*). qPCR analysis of ChIP DNA was performed as described previously from three independent control chromatin samples. Error bars are SEM. **B**) Signal peaks intensities of the same genomic loci were extracted from one of the NimbleGen ChIP-chip data sets. ChIP/Inputs ratio signals from 7 consecutive probes spanning the center of the region defined by each primer set used in the qPCR experiment were grouped an the average and SEM signal calculated. Error bars are SEM. Acetylation profiles for these unique genomic loci reported by these two methods show very high correlation as measured by the Pearson's correlation coefficient (r = 0.849, P = 0.0019).
(TIF)

Figure S2 FDR plots of drug induced acetylation peaks. Shown are false discovery rate (FDR) plots of all peaks identified in the difference arrays of histone acetylation between benzyl alcohol treated flies and control flies (top) or ethanol treated flies and controls flies (bottom). Only gene associated with peaks that have an FDR<0.05 were used in this study (shaded area).
(TIF)

Table S1 Treatment conditions used for co-expression clustering.
(DOC)

Table S2 Primers used in validation of ChIP-chip data by qPCR.
(DOC)

Table S3 Primers used in gene-expression analysis of candidate genes by qPCR.
(DOC)
