# Supplementary figures and images for: Alcohol-Induced Histone Acetylation Reveals a Gene Network Involved in Alcohol Tolerance
Source: PLoS Genet. 2013 Dec 12;9(12):e1003986. doi: 10.1371/journal.pgen.1003986 (PMC3861128; doi:10.1371/journal.pgen.1003986)

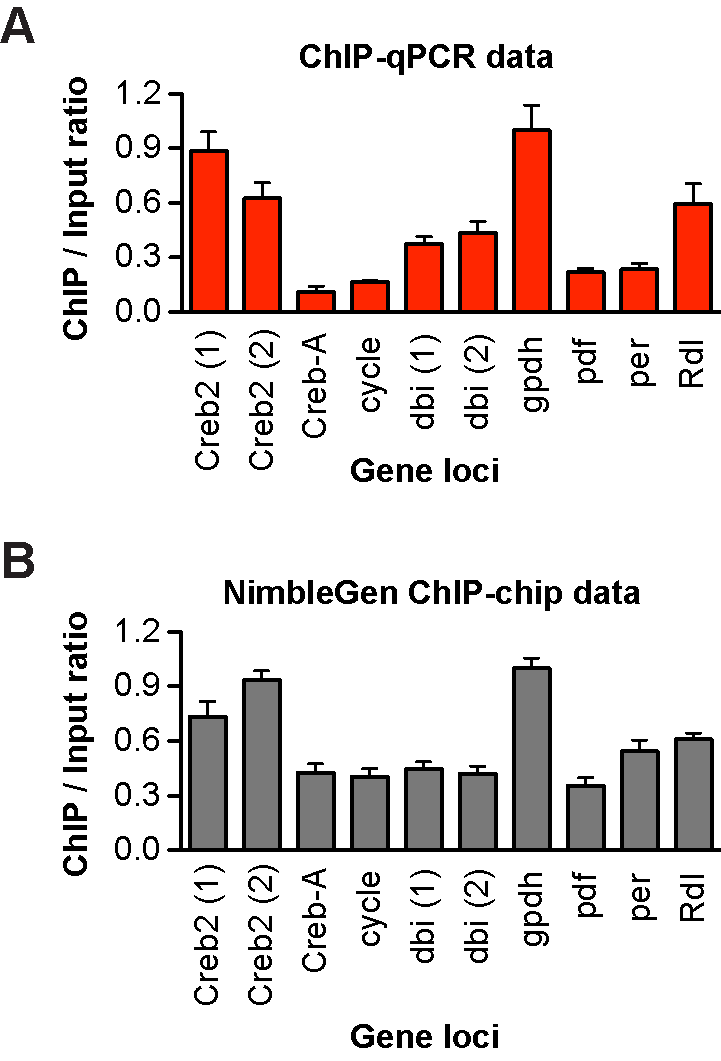

Supplement: Figure S1 — qPCR validation of NimbleGen ChIP-chip data. Shown are ChIP/Input ratios for 10 different gene loci as measured by qPCR or a NimbleGen DNA tilling array. A) Primer sets were designed for 10 unique loci across the genome mapping to the promoter region of 8 different genes (Creb2, CrebA, Cyc, dbi, gpdh, pdf, per and Rdl). qPCR analysis of ChIP DNA was performed as described previously from three independent control chromatin samples. Error bars are SEM. B) Signal peaks intensities of the same genomic loci were extracted from one of the NimbleGen ChIP-chip data sets. ChIP/Inputs ratio signals from 7 consecutive probes spanning the center of the region defined by each primer set used in the qPCR experiment were grouped an the average and SEM signal calculated. Error bars are SEM. Acetylation profiles for these unique genomic loci reported by these two methods show very high correlation as measured by the Pearson's correlation coefficient (r = 0.849, P = 0.0019). (TIF) [file pgen.1003986.s003.tif]

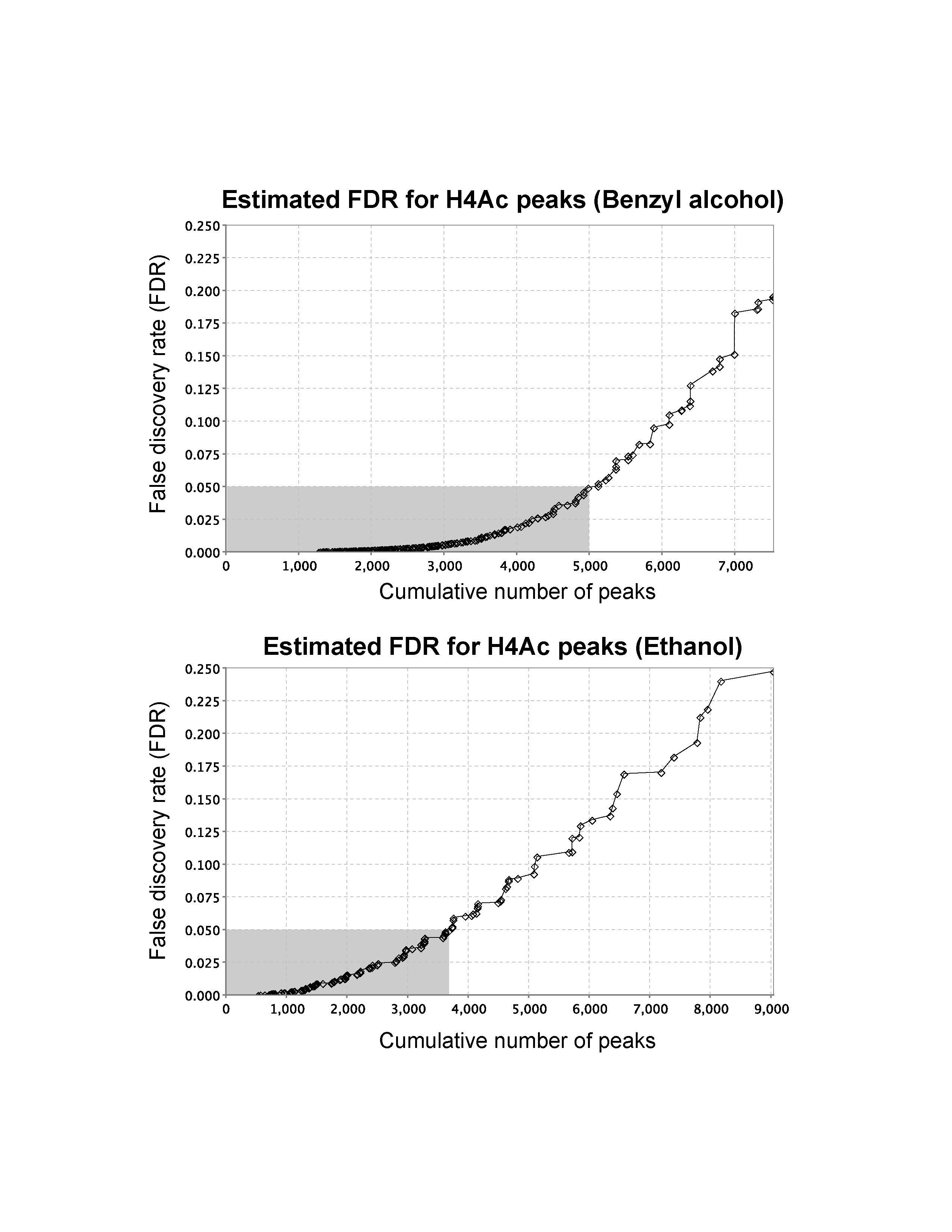

Supplement: Figure S2 — FDR plots of drug induced acetylation peaks. Shown are false discovery rate (FDR) plots of all peaks identified in the difference arrays of histone acetylation between benzyl alcohol treated flies and control flies (top) or ethanol treated flies and controls flies (bottom). Only gene associated with peaks that have an FDR<0.05 were used in this study (shaded area). (TIF) [file pgen.1003986.s004.tif]
