# Supplementary material for: Alcohol-Induced Histone Acetylation Reveals a Gene Network Involved in Alcohol Tolerance
Source: PLoS Genet. 2013 Dec 12;9(12):e1003986. doi: 10.1371/journal.pgen.1003986 (PMC3861128; doi:10.1371/journal.pgen.1003986)
Supplement: Table S1 — Treatment conditions used for co-expression clustering. (DOC) [file pgen.1003986.s005.doc]

**Supporting Table S1:** Treatment conditions used for co-expression clustering.

| **#** | **Treatment** | **Dose** | **Developmental stage** |
| --- | --- | --- | --- |
| 1 | extended cold | n/a | 4-day adult |
| 2 | cold shock | n/a | 4-day adult |
| 3 | heat shock | n/a | 4-day adult |
| 4 | Cadmium | 50 mM, 6 hrs | larvae L3 |
| 5 | Cadmium | 50 mM, 12 hrs | larvae L3 |
| 6 | Cadmium | 50 mM, 48 hrs | 4-day adult |
| 7 | Cadmium | 100 mM, 48 hrs | 4-day adult |
| 8 | Copper | 0.5 mM, 12 hrs | larvae L3 |
| 9 | Copper | 15 mM, 48 hrs | 4-day adult |
| 10 | Zinc | 5 mM, 12 hrs | larvae L3 |
| 11 | Zinc | 4.5 mM, 48 hrs | 4-day adult |
| 12 | Ethanol | 2.5%, 3 hrs | larvae L3 |
| 13 | Ethanol | 5%, 3 hrs | larvae L3 |
| 14 | Ethanol | 10%, 3 hrs | larvae L3 |
| 15 | Caffeine | 1.5 mg/ml, 4 hrs | larvae L3 |
| 16 | Caffeine | 2.5 mg/ml, 48 hrs | 4-day adult |
| 17 | Caffeine | 25 mg/ml, 48 hrs | 4-day adult |
| 18 | Paraquat | 5 mM, 48 hrs | 4-day adult |
| 19 | Paraquat | 10 mM, 48 hrs | 4-day adult |
| 20 | Rotenone | 2 μg, 12 hrs | larvae L3 |
| 21 | Rotenone | 8 μg, 12 hrs | larvae L3 |

(n/a) Information not available.
