# Supplementary material for: Alcohol-Induced Histone Acetylation Reveals a Gene Network Involved in Alcohol Tolerance
Source: PLoS Genet. 2013 Dec 12;9(12):e1003986. doi: 10.1371/journal.pgen.1003986 (PMC3861128; doi:10.1371/journal.pgen.1003986)
Supplement: Table S2 — Primers used in validation of ChIP-chip data by qPCR. (DOC) [file pgen.1003986.s006.doc]

**Supporting Table S2:** Primers used in validation of ChIP-chip data by qPCR.

| **Primer** | **Sequence (5'-3')** |
| --- | --- |
| Creb2-forward | GCGTCGCCGTTGGTTAGT |
| Creb2-reverse | TTTGGGAAGGCCGTCTGTT |
| CrebA-forward | GAGGAAACATCAATTCATCATATCCTT |
| CrebA-reverse | CAGAAAACCTCGAACAATTGATACTTT |
| Cyc-forward | GGGACCAAGAAACCAGATGTCTT |
| Cyc-reverse | TGATACATTTTATCCAAGCATATCGTT |
| dbi-forward | TGTTATCCGTAGGCCTGCATT |
| dbi-reverse | TCTCAGTGGATCGGGAAGCT |
| gpdh-forward | GCATACCTTGATCTTGGCCGT |
| gpdh-reverse | GCCCTGAAAAGTGCAAGAAG |
| pdf-forward | TCCCGTACAAATATCGCTGGAC |
| pdf-reverse | GTTATGCAGTGTTTTATGTGCCAAC |
| per-forward | GGCATTCCAATTACTTGCTTTTGT |
| per-reverse | TGAGATACTCGGACCCAAACATT |
| Rdl-forward | AAGTAACCTGATTACGGGCAGAGA |
| Rdl-reverse | TGGTTCTGAATCGGATTCCTTT |
