# Supplementary material for: Alcohol-Induced Histone Acetylation Reveals a Gene Network Involved in Alcohol Tolerance
Source: PLoS Genet. 2013 Dec 12;9(12):e1003986. doi: 10.1371/journal.pgen.1003986 (PMC3861128; doi:10.1371/journal.pgen.1003986)
Supplement: Table S3 — Primers used in gene-expression analysis of candidate genes by qPCR. (DOC) [file pgen.1003986.s007.doc]

**Supporting Table S3:** Primers used in gene expression analysis of candidate genes by qPCR.

| **Primer** | **Sequence (5'-3')** |
| --- | --- |
| para-forward | GAGCCCCAAGTACTATTTCCAG |
| para-reverse | GTCCCAGTTCCAATAGCGATAG |
| eag-forward | GTATCGGTTCCCTGTTCAGTG |
| eag-reverse | CCAGGTAGCGATCCAGTTTTC |
| brp-forward | CGAGAAGCTGGACAAGACG |
| brp-reverse | CGAATGACTCCGACTCGTATTG |
| Teh2-forward | CTCGTGGGAGAACAATCTGTAC |
| Teh2-reverse | CAGTACCAATAGCTGAGCACC |
| pum-forward | GCCCAGATGCCGTACTATG |
| pum-reverse | CGTTCCCTGTTGCGGAATC |
| nej-forward | AGAAGGAGTTTATGGATGACAGC |
| nej-reverse | GTTCACATTCTTGCCCTTGC |
| Cyp1-forward | GAGAAGGGATTCGGGTACAAG |
| Cyp1-reverse | TGTTGCCGTAGATGGACTTG |
